# Cannabis and Pain Treatment—A Review of the Clinical Utility and a Practical Approach in Light of Uncertainty

**DOI:** 10.5041/RMMJ.10385

**Published:** 2020-01-30

**Authors:** Simon Vulfsons, Amir Minerbi, Tali Sahar

**Affiliations:** 1 Institute for Pain Medicine, Rambam Health Care Campus, Haifa, Israel; 2 Rappaport School of Medicine, Technion Institute of Technology, Haifa, Israel; 3 Alan Edwards Centre for Research on Pain, Montreal, Quebec, Canada; 4 Pain Relief Unit, Department of Anesthesia, Hadassah Medical Center, Jerusalem, Israel; 5 Supportive Care & Pain Relief Clinic, Clalit Health Services, Jerusalem District, Israel; 6 Department of Family Medicine, Hebrew University of Jerusalem, Jerusalem, Israel

**Keywords:** Beneficial effects, cannabis, cannabis for therapeutic purposes, medical cannabis, review, side effects

## Abstract

Over the past decade the phenomenon of cannabis as a legitimate form of treatment for pain has overwhelmed the medical community, especially in the field of pain. From a status of a schedule 1 substance having no currently accepted medical use and being considered to have high potential for abuse, its use has mushroomed to over 50,000 legal medical users per year in Israel alone. There appear to be many reasons behind this phenomenon—medical, sociological, and economical. Thus, what is cannabis? An abusive substance or a medication? Should it be incorporated into current biomedical practice, and how should it be administered? Finally, what is the evidence for the beneficial and detrimental effects of cannabis? This article reviews and discusses the current literature regarding the beneficial and the detrimental effects of medical cannabis in the treatment of pain. We further discuss the problems and challenges facing the medical community in this domain and offer a practical approach to deal with these challenges.

## INTRODUCTION

Over the past decade the phenomenon of cannabis as a legitimate form of treatment for pain has overwhelmed the medical community, especially in the field of pain. From a status of a schedule 1 substance,^1^ having no currently accepted medical use and being considered to have high potential for abuse, its use has mushroomed to over 50,000 legal medical users per year in Israel alone (Ministry of Health: personal communication). There appear to be many reasons behind this phenomenon. Opiate medications, used and abused over the last two decades, have brought about a crisis in treatment—the opioid overdose crisis.^2^–^4^ In a nutshell, prescription opiates have brought about a huge rise in all-cause mortality in the United States, leading to over 130 deaths per day, with an estimated cost of 78.5 billion dollars in healthcare, lost productivity, addiction treatment, and criminal justice involvement.^5^,^6^ In October 2017 the opioid crisis was declared by presidential decree to be a public health emergency in the United States.^7^ This crisis in prescription medications for the treatment of pain has arisen on the tail of a previous crisis: that of the adverse effects of non-steroidal anti-inflammatory drugs (NSAIDs).^8^ The NSAIDs crisis became most apparent after the article by Wolfe et al. in 1999.^9^ Thus, from a medical point of view, two mainstays of the pharmacological treatment of chronic pain, NSAIDs and opiates, are now deemed inappropriate and dangerous, leaving a vacuum which is being filled by cannabis.

Other reasons behind the role that cannabis is taking in the treatment of pain include massive campaigns for the decriminalization and legalization of cannabis, as well as the so-called “medicalization” of cannabis.^10^,^11^ Bostwick, in a seminal paper in 2012, described the blurred boundaries between cannabis as a medical agent and cannabis as a recreational one.^12^ In fact the boundaries between recreational and “medical” cannabis use is constantly negotiated between stakeholders as part of the regulatory boundary-work, depending on each stakeholder’s particular interests.^13^ The medicalization of cannabis is contested by stakeholders, and in Israel this is an ongoing process.^14^ The Israeli government authority for cannabis has opted for a biomedical approach, viewing cannabis as a biological substance that can be incorporated into medical practice, although marked uncertainty exists concerning optimal strains, concentrations, doses, and modes of delivery. In contrast to the Israeli cannabis authority, physicians’ attitudes and views, in Israel, are far less accepting towards this substance.^15^ An earlier study suggested that Israeli physicians exhibited partial acceptance of medical cannabis as a therapeutic agent,^16^ while a later in-depth study has shown a conflicted view of physicians’ acceptance of medical cannabis.^17^ Public opinion can be swayed by media coverage, as evidenced in a study published in 2015 in which it was found that 69% of news articles in the three major daily newspapers in Israel framed cannabis as a medicine.^18^

Thus, what is cannabis? An abusive substance or a medication? Should it be incorporated into current biomedical practice, and how should it be administered? Finally, what is the evidence for the beneficial and detrimental effects of cannabis?

In this article we will discuss the beneficial effects, the detrimental effects, and the problems and challenges facing the medical community concerning cannabis in the treatment of pain.

## CANNABIS: BENEFICIAL EFFECTS IN PAIN TREATMENT

The classification of pain dictates that we approach the question of beneficial effects by subgrouping pain into various types. Acute pain, often considered to be a symptom of tissue damage and nociceptive activation, is very different from chronic pain, often considered a disease in its own right.^19^ Thus, we will attempt to describe the evidence of different subgroups of pain.

### Acute Pain

The response of patients, suffering from acute pain, to pharmaceutical and herbal cannabinoids has been explored both in the clinical setting and in laboratory conditions on healthy volunteers.

Pain reduction was examined in a trial of 40 women undergoing abdominal hysterectomy and receiving a single dose of either 5 mg of δ-9-tetrahydrocannabinol in capsule form or placebo. No analgesic effect was observed in either group.^20^ In contrast, escalating doses of 5, 10, and 15 mg cannabis extract were used for post-surgical patients after patient-controlled analgesia cessation with dose response for decreasing pain intensity at rest, increasing sedation, and more adverse events.^21^ In an experimental trial of 18 healthy volunteers, oral cannabis extract or placebo were administered after induction of pain either in a sunburn model or an intradermal capsaicin injection model. No pain reduction was found in either the active or placebo medications.^22^ Other synthetic cannabinoids have also been tested for pain reduction on the model of postoperative pain. Forty-one patients underwent a double-blinded, randomized, placebo-controlled, parallel group trial post-surgery with nabilone, an oral synthetic tetrahydrocannabinol (THC) analogue with either 1 mg or 2 mg nabilone, ketoprofen 50 mg, or placebo. There were four groups altogether. Outcome measures were pain scores, morphine consumption, and emesis. There was no difference in pain scores between the groups. In fact, the higher dose of nabilone 2 mg was associated with increased pain at rest.^23^

In summary, it appears that cannabis is not an effective analgesic agent in the acute pain setting.

### Chronic Pain

#### Neuropathic and central pain

In a meta-analysis of cannabis-based treatments for neuropathic and multiple sclerosis (MS)-related pain, 7 randomized, double-blinded, placebo-controlled trials were included (*n*=298 patients).^24^ The overall quality of the studies was very good. In general, the overall reductions in pain were in excess of 1.5 points on an 11-point scale and were all statistically significant. The difference in effect size in comparison to placebo was 0.8. This difference, however, is considered barely clinically significant.^25^,^26^

In a systematic review of cannabinoids for the treatment of non-cancer pain, 18 trials published between the years of 2003 and 2010 involving 766 participants were included.^27^ The quality of the trials was good, and in 15 of the 18 trials there was a significant analgesic effect for the cannabinoid being tested. Four of the trials examined the effect of smoked cannabis on neuropathic pain, all reporting positive effects with minimal or no serious adverse effects. The mean treatment duration was only 8.5 days. Seven trials examined the effects of oromucosal extracts of cannabis-based medicine. Five trials examined the effect on participants with neuropathic pain, and four of these reported positive analgesic effects that were generally modest. Nabilone 2 mg has been found to be as effective as dihydrocodeine 240 mg for patients with neuropathic pain.^28^ Dronabinol 10 mg has been found to be modestly effective for central pain in MS.^29^

In an update of their previous systematic review of randomized controlled trials (RCTs) for cannabinoids for the treatment of chronic non-cancer pain, Lynch and Ware added 11 trials that met the inclusion criteria.^30^ They included randomized controlled trials published from 2010 to 2014 involving 1,185 subjects. Of these studies, seven demonstrated significant analgesic effects. Taken with the original systematic review by the same authors, 22 of 29 RCTs have demonstrated that cannabinoids demonstrate a modest analgesic effect and are safe in the management of chronic pain.^27^ It should be noted that of these 29 studies the following modes of delivery were explored: smoked cannabis (6 trials), oromucosal and oral cannabis extract (11 trials), nabilone (8 trials), dronabilone (2 trials), THC-11-oic acid analogue (2 trials), and fatty acid amide hydrolase inhibitor (FAAH) inhibitor (1 trial). In summary, the 6 smoked cannabis trials all showed a positive analgesic response, 10 of the 11 oromucosal and oral extract trials showed a positive analgesic response, 6 of the 8 trials examining the analgesic effects of nabilone were positive, both dronabinol trials had positive analgesic effect, and neither the THC-11-oic acid analogue nor the FAAH inhibitor trials showed lasting positive analgesic effects. All articles declared modest analgesic effects at best.

#### Musculoskeletal pain

Chronic musculoskeletal pain affects a large percentage of the population, including arthritis, back pain, and post-trauma/surgical pain.^31^–^33^ A recent critical review of the literature was published addressing this chronic pain population.^34^ A total of 118 trials were included in the study, of which 33 were considered as covering “core orthopedic topics” (arthritis, back pain, trauma-related and postoperative pain). A large proportion of studies were observational, and there were very few level I randomized control studies in the core orthopedic topics. In summary the authors concluded that there is little high-quality evidence for effective analgesia in core orthopedic musculoskeletal pain, but the “best available” evidence suggests cannabis may be effective for managing arthritis pain, back pain, and trauma-related pain, although the quality of the evidence is poor.

#### Fibromyalgia

A Cochrane systematic review published in 2016 on the use of cannabinoids to treat fibromyalgia found only two studies of at least four weeks’ duration that compared cannabinoids to either placebo or amitriptyline.^35^ The cannabinoid studied was nabilone 1 mg per day at bedtime. The authors found no convincing, unbiased, high-quality evidence suggesting that nabilone is of value in treating people with fibromyalgia. Furthermore, the tolerability of nabilone was low in people with fibromyalgia.^36^,^37^

Worthy of mention is a study published in 2019 where four varieties of pharmaceutical grade cannabis were administered by single shot vapor to patients suffering from fibromyalgia. The rigorous design of the study explored the effect of: (1) high THC content (Bedrocan: THC 22.4 mg, cannabidiol [CBD] 1 mg); (2) balanced THC/CBD content (Bediol: THC 13.4 mg, CBD 17.8 mg); (3) high CBD content (Bedrolite: CBD 18.4 mg, THC 1 mg); and (4) placebo.^38^ None of the treatments had an effect greater than placebo on spontaneous or electrical evoked pain responses, although more subjects receiving Bediol (balanced THC/CBD content) displayed a 30% decrease in pain scores compared to placebo (90% versus 55% of patients, *P*=0.01), with spontaneous pain scores correlating with the magnitude of drug high. It is impossible to extrapolate the long-term effect of vaporized cannabis on fibromyalgia patients.

#### Symptoms accompanying chronic pain

Chronic pain patients carry a high prevalence of accompanying symptoms such as depression, sleep disturbances, fatigue, decrease in daily function, cognitive dysfunction, and more.^39^ A few reports in the literature have described the effect of cannabinoids on quality of life symptoms in chronic pain patients. In a prospective open label study, 274 patients were followed for six months. Only 176 patients completed the study. Mild improvements were found in pain symptom, severity, and interference scores.^40^ In a cross-sectional survey study, 56 fibromyalgia patients completed the study, of whom 28 (50%) were cannabis users, mainly smokers. The cannabis had been used for up to three years. In the quality of life scales there was a mild increase in the mental health component summary score of the SF-36 questionnaire, but no difference was found between cannabis users and non-users for the fibromyalgia impact questionnaire or the Pittsburg sleep quality index.^41^

Thus, it is safe to say that quality of life indices improve only slightly, if at all, for fibromyalgia patients using cannabis.

In summary of the beneficial effects of cannabis and cannabis-related substances in the treatment of pain, it appears that cannabis is not effective in the treatment of acute pain, has mild beneficial effects for neuropathic pain, and may be effective in core orthopedic musculoskeletal pain. In general, the level of studies of cannabinoids for neuropathic pain were good, while the studies for musculoskeletal pain were poor.

## CANNABIS: ADVERSE EFFECTS, DRUG–DRUG INTERACTIONS, AND SPECIAL CONSIDERATIONS

Since the cannabis plant (*Cannabis sativa L.*) contains more than 100 phytocannabinoids and since cannabinoid receptors are found throughout the human body, any discussion about medical cannabis is bound to be a simplified description of a highly complex system that involves multivariate interactions.

It has been suggested that the complex biosystem of cannabis could explain both the high number needed to treat (NNT; since it takes time for the patients to find their ideal treatment) and the low number needed to harm (NNH; since 20%–80% of the patients experience side effects).^25^ Fortunately, most side effects are transient and generally well tolerated,^38^,^42^ yet some adverse effects may be detrimental, especially in vulnerable populations (Table 1).^38^

**Table 1:** A Literature-based Summary of Medical Cannabis-related Adverse Events According to the Involved Biosystems.^27^,^30^,^35^–^38^,^40^–^58^

Biosystem	Adverse Events		
CNS-Related: Neurological and Cognitive (References: 27,30,35–38,40–51,56,58)	Drowsiness Dizziness Heaviness Vertigo Confusion Fatigue/lethargy/somnolence Impaired attention/lack of concentration/mental clouding	Disorientation Impaired memory Impaired psychomotor skills/incoordination/ataxia/higher rate of MVAs Numbness Slurred speech	Blurred vision/diplopia Impaired hearing/tinnitus Headaches/migraine Hyperalgesia/increased pain
CNS-Related: Psychological (References: 27,30,35–38,40–49,52,54,58)	Restlessness/anxiety/nervousness Depressed mood/depression/dysphoria Euphoria/feeling high Feeling abnormal	Confusion Disinterest Dissociation Hallucinations Hyperactivity	Nightmares/weird dreams Paranoia Racing thoughts Psychosis
Cardiovascular and ANS (References: 27,30,35–38,40–46,53,55,58)	Myocardial infarction CVA Abnormal heart rate Cardiac disorders Hypertension	Hypotension/orthostatic/hypotension Palpitations Tachycardia	Sweating/diaphoresis/hot flushes/facial flushes Red eyes/dry eyes/rash/dry skin
GI and Metabolism (References: 27,30,35–38,40–46,53,58)	Nausea/vomiting/hyperemesis Loss of appetite/increased appetite/thirst Abdominal discomfort/pain Anorexia	Bad taste Constipation Diarrhea Dry mouth Dyspepsia/epigastric distress	Glossodynia/hypoesthesia Oral/mucosal ulceration/irritation/sore mouth
Other Mechanisms (References: 27,30,38,40–46,48,54,56–58)	Infections and infestations: aspergillosis/pharyngitis/URTI/dyspnea Respiratory: cough Hoarseness	Tobacco-related (concomitant use with tobacco): chronic lung disease Endocrine: decreased LH/FSH/GH/prolactin	Immunological: aggravated MS symptoms/MS relapse Drug-drug interactions: elevated liver enzymes Hepatobiliary disorders

ANS, autonomic nervous system; CNS, central nervous system; CVA, cerebrovascular accident: FSH, follicle-stimulating hormone; GH, growth hormone; GI, gastrointestinal; LH, luteinizing hormone; MS, multiple sclerosis; MVA, motor vehicle accident; URTI, upper respiratory tract infection.

Several systematic reviews of RCTs and of safety studies have examined the adverse events following short-term treatment with medical cannabis.^30^,^40^–^42^ Unfortunately, when systematically reviewed, many of these trials are judged as low-quality studies with a high risk of bias.^35^,^44^ Disappointingly, many of the reviewed RCTs lacked quantifiable adverse event data or did not report any data regarding adverse events.^43^ Authors agree that the rate of adverse events is likely underreported.^42^,^43^ This may change in the future, since it appears that newer studies are of better quality.^30^ Furthermore, the cannabis and cannabinoid preparations studied are diverse and have substantial pharmacokinetic differences, therefore care should be taken when citing or drawing conclusions from these studies.^30^

The consequences of long-term treatment with medical cannabis have not been fully examined.^46^ Most of the RCTs with medical cannabis were of very short duration, generally several days.^47^ Longer-duration studies rarely lasted more than four weeks. This may lead to confusion for clinicians as well as patients and policymakers^47^; medical cannabis is currently prescribed for much longer periods, from several months to many years and even for lifetime.

Therefore, the literature regarding long-term adverse effects is based on experience with long-term recreational use of cannabis, rather than on RCTs of medical cannabis.^48^ Such studies suggest that, in the long run, cannabis can cause dependency, as well as cognitive changes,^49^ anatomical brain damage,^50^,^51^ and psychosis.^52^

Side effects of short-term treatment are very common, but in the most part these are not serious.^43^ They may differ from person to person, and even the same person may experience different side effects at different times. Many factors influence the probability and the severity of adverse events, such as: type of cannabinoid preparation^47^; the mode of administration^45^; the patient’s attitude and expectations^53^ as well as age^54^,^55^; and drug–drug interactions.^56^,^58^

In contrast to prescription medications, cannabis and cannabinoids are not a single agent and thus have multiple and diverse side effects. Moreover, certain side effects can have a conflicting effect. For example, cannabis can cause either hypotension or hypertension, weight gain or weight loss, euphoria or anxiety. This has been attributed by some researchers to the non-linear concentration-effect curve of cannabis. For some effects of endocannabinoids and cannabinoids the concentration-effects curve seems to have an inverted U shape: when a maximum effect is reached, further increases of concentration decrease the effect or even cause an opposite effect due to desensitization and downregulation of receptors. 58

Therefore, we present these side effects according to the biological mechanisms that drive them, hoping that it will serve as a simple scheme for patients and clinicians.

Practically, if a patient experiences symptoms in any of these systems while treated with medical cannabis, the clinician may suggest reducing the doses, changing concentrations or modes of administration, and, in severe cases, considering discontinuation.

A safe way to reduce or avoid side effects is gradual titration of low doses of cannabinoids (“start low and go slow”).^58^–^60^

There is evidence that higher doses and higher initial cannabinoid doses, as well as fast titration, can increase adverse events, with no symptomatic benefits (sometimes related to as an upside down “U”-shaped response curve).^58^,^59^

### Drug–Drug Interactions

Both THC and CBD are metabolized by the liver cytochrome P450 (CYP-450) system. Cannabinoids may act as substrates, inhibitors, or inducers of various CYP-450 isoforms. Moreover, medical cannabis is often prescribed for people with complicated medical conditions^61^ and is often co-administered with various medications. Accordingly, potential drug–drug interactions (DDI) of cannabinoids with other drugs or herbs are multiple and often overlooked, by both prescribers and patients.

Several reviews^56^,^62^ have examined this topic and found that DDIs are very common not only with formulations containing THC, but even with pure CBD,^56^ which has mistakenly gained reputation as an inert compound. Drug–drug interactions are often dose-dependent and possibly less predictable in edible CBD products or in elderly patients.^55^

A professional committee of the Pharmaceutical Society of Israel^57^ has summarized the data from several pharmaceutical databases and created a table of the main DDI with dozens of medications and herbs according to the underlying mechanisms of interaction. Brown and Winterstein^56^ also offer such tables in their review. We suggest that healthcare providers carefully follow patients that are concomitantly taking other medications or herbs, surveying for possible DDIs that are published in pharmaceutical data bases. Special attention should be given to medications with neurological, respiratory, or psychiatric effects.

### Pregnancy

There is growing concern regarding the use of products that contain THC and/or CBD during pregnancy and breastfeeding. Recently the United States Food and Drug Administration (FDA) strongly advised against the use of CBD, THC, or marijuana, in any form, during pregnancy and while breastfeeding.^63^ The FDA’s recommendation is based on that of the US Surgeon General’s Advisory.^64^ Cannabis and cannabinoids in pregnancy are associated with adverse outcomes, including lower birth weight.^65^ Moreover, exogenous cannabinoids may interfere with the delicate balance of the endocannabinoid system regulation of the female reproductive system.^66^

## DISCUSSION

**Figure 1:**
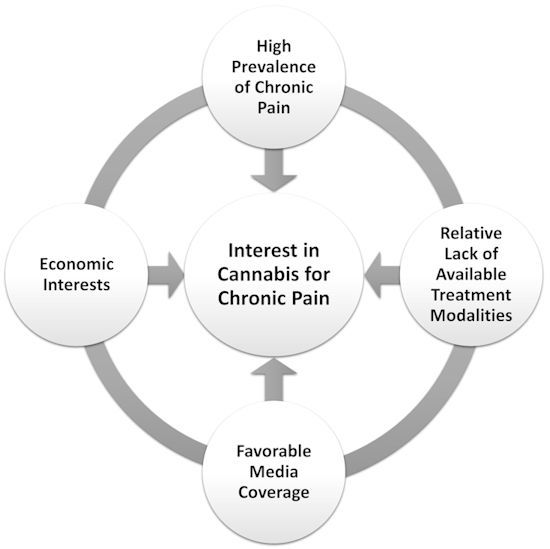
A Schematic Representation of Putative Factors Contributing to Rising Public Interest in Medicinal Cannabis for Chronic Pain.

Interest in medicinal use of cannabis and cannabinoids for chronic pain is increasing worldwide, and the number of patients who use it either by prescription or as self-medication is rising rapidly.^67^,^68^ In our clinical practice, we encounter patients seeking treatment with cannabis on a daily basis. Several factors may contribute to this observed rise in demand (Figure 1): first, an unmet need for pain relief. Chronic pain is an exceedingly prevalent medical problem, estimated to affect about one-fifth of the world’s adult population.^69^ Treatment modalities for chronic pain include pharmaceutical, physical, psychological, and invasive measures, as well as complementary and alternative medicine.^70^ Even with best treatment, many patients continue to suffer from considerable symptoms, negatively affecting their quality of life. Effective pharmaceutical treatment options are rather limited, more so in light of appreciation of the adverse effects of long-term use of anti-inflammatory and opioid medications.^71^,^72^ Second, public interest is fueled by favorable coverage of the medical virtues of cannabis by the mass media as well as in online resources,^73^ contributing to the common perception of cannabis as a natural panacea, both safe and effective for a multitude of ailments.^18^ Finally, the increasing interest in medical cannabis is fueled by massive economic interests of a rapidly growing cannabis industry,^74^ which is heavily invested in the medicalization of cannabis products.

This overwhelming demand for medical cannabis stands in contradiction to the paucity of evidence of its efficacy and safety. Thus, while some studies show a mild analgesic effect of cannabinoids in neuropathic pain and central pain, overall the available evidence on the safety and efficacy of cannabis for chronic pain is inconclusive. Recent meta-analyses suggest that many patients have to be treated in order for a few to benefit, while the risks of harm are significant.^75^ This could explain the reported discrepancy between the perception of the utility of cannabis among clinicians as compared to among patients.^15^,^76^ Nevertheless, despite the apparent lack of efficacy of cannabis for chronic pain as reflected in the medical literature, in our experience individual patients occasionally seem to draw considerable benefit from its use. Given the modest effect size on pain reduction, some have argued that the benefits could lie in other domains, such as positive effects on anxiety, sleep, and overall well-being. However, results of studies on the effects of cannabis in these domains do not support this notion.^77^

The discrepancy between patients’ opinion on the utility of cannabis for chronic pain and the lack of conclusive evidence of efficacy and safety is a common source of conflict in clinical practice. As clinical guidelines are lacking and evidence is scanty, the physician must make a decision under uncertainty. We propose a practical clinical approach to making a decision on cannabis treatment^55^:

1. **Consider the indication:** Is cannabis potentially effective for the patient’s condition? Does the patient present with accompanying symptoms which may benefit from the use of cannabinoids (i.e. insomnia, cachexia)?
2. **Consider other treatment modalities:** Are there any other potentially effective treatment modalities, which have not yet been explored? Consider pharmaceutical, physical, psychological, and invasive options. This is not to say that cannabis should only be considered as a last-resort treatment, but, given the uncertainty of its efficacy and adverse effects profile, other, more established options may be considered first.
3. **Consider contra-indications:** In particular, patients should be assessed for: (a) psychiatric comorbidities—exclude patients deemed at risk for psychosis, mania, and suicidal tendency; (b) cardiovascular disease both established and with risk factors—while this is not an absolute contraindication, these patients warrant particular consideration before cannabis can be offered to them; (c) cognitive impairment—patients with limited cognitive reserve can suffer a significant impairment of function and independence if treated with cannabinoids; and (d) frailty, polypharmacy, and problems of gait and balance—these should be evaluated and addressed. To sum up, we suggest having an open discussion of the pros and cons of medical cannabinoids with the patient, raising possible cardiovascular, psychiatric, and cognitive effects, as well as potential effects on driving.
4. **Assess benefit–risk profile:** The benefit–risk function is individual and varies between patients and physicians, corresponding to their beliefs and values. It should also be noted that individuals receiving palliative care may sometimes be willing to take more risks in order to achieve improved quality of life, compared to individuals who are deemed to have longer life expectancies.
5. **Begin treatment:** We suggest defining the initial prescription of medical cannabis as a treatment trial.
6. **Re-assessment:** Following the initiation of a cannabinoid, a short-interval follow-up visit or telephone call is recommended. The treatment efficacy is evaluated for the various symptoms, and side effects are documented. We recommend very close and supportive guidance for patients receiving medical cannabis.^60^

The current state of uncertainty and lack of evidence calls for caution in the use of cannabis in clinical practice. The way for the full integration of cannabis as a pharmaceutical agent is still long: cannabis preparations will have to be standardized, such that the content of active molecules and their bioavailability is clear and consistent. In parallel, the entourage effect should be further studied. Furthermore, high-quality evidence from clinical trials and clinical registries is needed.^78^ Clinical registries should include options for clinicians and patients to report side effects and emergency room visits.

Until then, a cautious approach is recommended, weighing the evidence and individual patients’ needs, without succumbing to public pressure.

## Abbreviations

CBD: cannabidiol
DDI: drug–drug interactions
MS: multiple sclerosis
NSAID: non-steroidal anti-inflammatory drugs
NNH: number needed to harm
NNT: number needed to treat
RCT: randomized controlled trials
THC: tetrahydrocannabinol.
